# Supplementary material for: A morphological transformation in respiratory syncytial virus leads to enhanced complement deposition
Source: eLife. 2021 Sep 29;10:e70575. doi: 10.7554/eLife.70575 (PMC8480979; doi:10.7554/eLife.70575)
Supplement: Supplementary file 1. [file elife-70575-supp1.pdf]

RSV sequences

RSV A2 (Line 19F):

NS1-IRES-mTagBFP2/NS2/N/P/M/SH/G(**ybbR**)/(**G5**)F/M2/L

ACGCGAAAAAATGCGTACAACAAACTTGCATAAACCAAAAAAATGGGGCAAATAAGAATTTGATAAGTACCACTTAAATTTAACTCCCTTGGTTAGAGATGGGCAGCAATTC
ATTGAGTATGATAAAAGTTAGATTACAAAATTTGTTTGACAATGATGAAGTAGCATTGTTAAAAATAACATGCTATACTGATAAATTAATACATTTAACTAACGCTTTGGCT
AAGGCAGTGATACATACAATCAAATTGAATGGCATTGTGTTTGTGCATGTTATTACAAGTAGTGATATTTGCCCTAATAATAATATTGTAGTAAAATCCAATTTCACAACAA
TGCCAGTACTACAAAATGGAGGTTATATATGGGAAATGATGGAATTAACACATTGCTCTCAACCTAATGGTCTACTAGATGACAATTGTGAAATTAAATTCTCCAAAAAACT
AAGTGATTCAACAATGACCAATTATATGAATCAATTATCTGAATTACTTGGATTTGATCTTAATCCATAACGGTTTCCCTCTAGCGGGATCAATTCCGCCCCCCCCCCCTAA
CGTTACTGGCCGAAGCCGCTTGGAATAAGGCCGGTGTGCGTTTGTCTATATGTTATTTTCCACCATATTGCCGTCTTTTGGCAATGTGAGGGCCCGGAAACCTGGCCCTGTC
TTCTTGACGAGCATTCCTAGGGGTCTTTCCCCTCTCGCCAAAGGAATGCAAGGTCTGTTGAATGTCGTGAAGGAAGCAGTTCCTCTGGAAGCTTCTTGAAGACAAACAACGT
CTGTAGCGACCCTTTGCAGGCAGCGGAACCCCCCACCTGGCGACAGGTGCCTCTGCGGCCAAAAGCCACGTGTATAAGATACACCTGCAAAGGCGGCACAACCCCAGTGCCA
CGTTGTGAGTTGGATAGTTGTGGAAAGAGTCAAATGGCTCTCCTCAAGCGTATTCAACAAGGGGCTGAAGGATGCCCAGAAGGTACCCCATTGTATGGGATCTGATCTGGGG
CCTCGGTGCACATGCTTTACATGTGTTTAGTCGAGGTTAAAAAAACGTCTAGGCCCCCCGAACCACGGGGACGTGGTTTTCCTTTGAAAAACACGATAATACCATGAGCGAG
CTGATTAAGGAGAACATGCACATGAAGCTGTACATGGAGGGCACCGTGGACAACCATCACTTCAAGTGCACATCCGAGGGCGAAGGCAAGCCCTACGAGGGCACCCAGACCA
TGAGAATCAAGGTGGTCGAGGGCGGCCCTCTCCCCTTCGCCTTCGACATCCTGGCTACTAGCTTCCTCTACGGCAGCAAGACCTTCATCAACCACACCCAGGGCATCCCCGA
CTTCTTCAAGCAGTCCTTCCCTGAGGGCTTCACATGGGAGAGAGTCACCACATACGAAGACGGGGGCGTGCTGACCGCTACCCAGGACACCAGCCTCCAGGACGGCTGCCTC
ATCTACAACGTCAAGATCAGAGGGGTGAACTTCACATCCAACGGCCCTGTGATGCAGAAGAAAACACTCGGCTGGGAGGCCTTCACCGAGACGCTGTACCCCGCTGACGGCG
GCCTGGAAGGCAGAAACGACATGGCCCTGAAGCTCGTGGGCGGGAGCCATCTGATCGCAAACGCCAAGACCACATATAGATCCAAGAAACCCGCTAAGAACCTCAAGATGCC
TGGCGTCTACTATGTGGACTACAGACTGGAAAGAATCAAGGAGGCCAACAACGAGACCTACGTCGAGCAGCACGAGGTGGCAGTGGCCAGATACTGCGACCTCCCTAGCAAA
CTGGGGCACAAGCTTAATTAAATTATAATTAATATCAACTAGCAAATCAATGTCACTAACACCATTAGTTAATATAAAACTTAACAGAAGACAAAAATGGGGCAAATAAATC
AATTCAGCCAACCCAACCATGGACACAACCCACAATGATAATACACCACAAAGACTGATGATCACAGACATGAGACCGTTGTCACTTGAGACCATAATAACATCACTAACCA
GAGACATCATAACACACAAATTTATATACTTGATAAATCATGAATGCATAGTGAGAAAACTTGATGAAAAACAGGCCACATTTACATTCCTGGTCAACTATGAAATGAAACT
ATTACACAAAGTAGGAAGCACTAAATATAAAAAAATATACTGAATACAACACAAAATATGGCACTTTCCCTATGCCAATATTCATCAATCATGATGGGTTCTTAGAATGCATT
GGCATTAAGCCTACAAAGCATACTCCCATAATATACAAGTATGATCTCAATCCATAAATTTCAACACAATATTCACACAATCTAAAACAACAACTCTATGCATAACTATACT
CCATAGTCCAGATGGAGCCTGAAAATTATAGTAATTTAAAATTAAGGAGAGATATAAGATAGAAGATGGGGCAAATACAAAGATGGCTCTTAGCAAAGTCAAGTTGAATGAT
ACACTCAACAAAGATCAACTTCTGTCATCCAGCAAATACACCATCCAACGGAGCACAGGAGATAGTATTGATACTCCTAATTATGATGTGCAGAAACACATCAATAAGTTAT
GTGGCATGTTATTAATCACAGAAGATGCTAATCATAAATTCACTGGGTTAATAGGTATGTTATATGCGATGTCTAGGTTAGGAAGAGAAGACACCATAAAAATACTCAGAGA
TGCGGGATATCATGTAAAAGCAAATGGAGTAGATGTAACAACACATCGTCAAGACATTAATGGAAAAGAAATGAAATTTGAAGTGTTAACATTGGCAAGCTTAACAACTGAA
ATTCAAATCAACATTGAGATAGAATCTAGAAAATCCTACAAAAAAATGCTAAAAGAAATGGGAGAGGTAGCTCCAGAATACAGGCATGACTCTCCTGATTGTGGGATGATAA
TATTATGTATAGCAGCATTAGTAATAACTAAATTAGCAGCAGGGGACAGATCTGGTCTTACAGCCGTGATTAGGAGAGCTAATAATGTCCTAAAAAATGAAATGAAACGTTA
CAAAGGCTTACTACCCAAGGACATAGCCAACAGCTTCTATGAAGTGTTTGAAAAACATCCCCACTTTATAGATGTTTTTGTTCATTTTGGTATAGCACAATCTTCTACCAGA
GGTGGCAGTAGAGTTGAAGGGATTTTTGCAGGATTGTTTATGAATGCCTATGGTGCAGGGCAAGTGATGTTACGGTGGGGAGTCTTAGCAAAATCAGTTAAAAATATTATGT
TAGGACATGCTAGTGTGCAAGCAGAAATGGAACAAGTTGTTGAGGTTTATGAATATGCCCAAAAATTGGGTGGTGAAGCAGGATTCTACCATATATTGAACAACCCAAAAGC
ATCATTATTATCTTTGACTCAATTTCCTCACTTCTCCAGTGTAGTATTAGGCAATGCTGCTGGCCTAGGCATAATGGGAGAGTACAGAGGTACACCGAGGAATCAAGATCTA
TATGATGCAGCAAAGGCATATGCTGAACAACTCAAAGAAAATGGTGTGATTAACTACAGTGTACTAGACTTGACAGCAGAAGAACTAGAGGCTATCAAACATCAGCTTAATC
CAAAAGATAATGATGTAGAGCTTTGAGTTAATAAAAAATGGGGCAAATAAATCATCATGGAAAAGTTTGCTCCTGAATTCCATGGAGAAGATGCAAACAACAGGGCTACTAA
ATTCCTAGAATCAATAAAGGGCAAATTCACATCACCCAAAGATCCCAAGAAAAAAGATAGTATCATATCTGTCAACTCAATAGATATAGAAGTAACCAAAGAAAGCCCTATA
ACATCAAATTCAACTATTATCAACCCAACAAATGAGACAGATGATACTGCAGGGAACAAGCCCAATTATCAAAGAAAACCTCTAGTAAGTTTCAAAGAAGACCCTACACCAA
GTGATAATCCCTTTTCTAAACTATACAAAGAAACCATAGAAACATTTGATAACAATGAAGAAGAATCCAGCTATTCATACGAAGAAATAAATGATCAGACAAACGATAATAT
AACAGCAAGATTAGATAGGATTGATGAAAAATTAAGTGAAATACTAGGAATGCTTCACACATTAGTAGTGGCAAGTGCAGGACCTACATCTGCTCGGGATGGTATAAGAGAT
GCCATGATTGGTTTAAGAGAAGAAATGATAGAAAAAATCAGAACTGAAGCATTAATGACCAATGACAGATTAGAAGCTATGGCAAGACTCAGGAATGAGGAAAGTGAAAAGA
TGGCAAAAGACACATCAGATGAAGTGTCTCTCAATCCAACATCAGAGAAATTGAACAACCTATTGGAAGGGAATGATAGTGACAATGATCTATCACTTGAAGATTTCTGATT
AGTTACCACTCTTCACATCAACACACAATACCAACAGAAGACCAACAAACTAACCAACCCAATCATCCAACCAAACATCCATCCGCCAATCAGCCAAACAGCCAACAAAACA
ACCAGCCAATCCAAAACTAACCACCCGGAAAAAATCTATAATATAGTTACAAAAAAAGGAAAGGGTGGGGCAAATATGGAAACATACGTGAACAAGCTTCACGAAGGCTCCA
CATACACAGCTGCTGTTCAATACAATGTCTTAGAAAAAGACGATGACCCTGCATCACTTACAATATGGGTGCCCATGTTCCAATCATCTATGCCAGCAGATTTACTTATAAA
AGAACTAGCTAATGTCAACATACTAGTGAAACAAATATCCACACCCAAGGGACCTTCACTAAGAGTCATGATAAACTCAAGAAGTGCAGTGCTAGCACAAATGCCCAGCAAA
TTTACCATATGCGCTAATGTGTCCTTGGATGAAAGAAGCAAACTAGCATATGATGTAACCACACCCTGTGAAATCAAGGCATGTAGTCTAACATGCCTAAAATCAAAAAATA
TGTTGACTACAGTTAAAGATCTCACTATGAAGACACTCAACCCTACACATGATATTATTGCTTTATGTGAATTTGAAAACATAGTAACATCAAAAAAAGTCATAATACCAAC
ATACCTAAGATCCATCAGTGTCAGAAATAAAGATCTGAACACACTTGAAAATATAACAACCACTGAATTCAAAAATGCTATCACAAATGCAAAAATCATCCCTTACTCAGGA
TTACTATTAGTCATCACAGTGACTGACAACAAAGGAGCATTCAAATACATAAAGCCACAAAGTCAATTCATAGTAGATCTTGGAGCTTACCTAGAAAAAGAAAGTATATATT
ATGTTACCACAAATTGGAAGCACACAGCTACACGATTTGCAATCAAACCCATGGAAGATTAACCTTTTTCCTCTACATCAGTGTGTTAATTCATACAAACTTTCTACCTACA
TTCTTCACTTCACCATCACAATCACAAACACTCTGTGGTTCAACCAATCAAACAAAACTTATCTGAAGTCCCAGATCATCCCAAGTCATTGTTTATCAGATCTAGTACTCAA
ATAAGTTAATAAAAAATATACACATGGGGCAAATAATCATTGGAGGAAATCCAACTAATCACAATATCTGTTAACATAGACAAGTCCACACACCATACAGAATCAACCAATG
GAAAATACATCCATAACAATAGAATTCTCAAGCAAATTCTGGCCTTACTTTACACTAATACACATGATCACAACAATAATCTCTTTGCTAATCATAATCTCCATCATGATTG
CAATACTAAACAAACTTTGTGAATATAACGTATTCCATAACAAAACCTTTGAGTTACCAAGAGCTCGAGTCAACACATAGCATTCATCAATCCAACAGCCCAAAACAGTAAC
CTTGCATTTAAAAATGAACAACCCCTACCTCTTTACAACACCTCATTAACATCCCACCATGCAAACCACTATCCATACTATAAAGTAGTTAATTAAAAATAGTCATAACAAT
GAACTAGGATATCAAGACTAACAATAACATTGGGGCAAATGCAAACATGTCCAAAAACAAGGACCAACGCACCGCTAAGACATTAGAAAGGACCTGGGACACTCTCAATCAT
TTATTATTCATATCATCGTGCTTATATAAGTTAAATCTTAAATCTGTAGCACAAATCACATTATCCATTCTGGCAATGATAATCTCAACTTCACTTATAATTGCAGCCATCA
TATTCATAGCCTCGGCAAACCACAAAGTCACACCAACAACTGCAATCATACAAGATGCAACAAGCCAGATCAAGAACACAACCCCAACATACCTCACCCAGAATCCTCAGCT
TGGAATCAGTCCCTCTAATCCGTCTGAAATTACATCACAAATCACCACCATACTAGCTTCAACAACACCAGGAGTCAAGTCAACCCTGCAATCCACAACAGTCAAGACCAAA
AACACAACAACAACTCAAACACAACCCAGCAAGCCCACCACAAAACAACGCCAAAACAAACCACCAAGCAAACCCAATAATGATTTTCACTTTGAAGTGTTCAACTTTGTAC
CCTGCAGCATATGCAGCAACAATCCAACCTGCTGGGCTATCTGCAAAAGAATACCAAACAAAAAACCAGGAAAGAAAACCACTACCAAGCCCACAAAAAAACCAACCCTCAA
GACAACCAAAAAAGATCCCAAACCTCAAACCACTAAATCAAAGGAAGTACCCACCACCAAGCCCACAGAAGAGCCAACCATCAACACCACCAAAACAAACATCATAACTACA
CTACTCACCTCCAACACCACAGGAAATCCAGAACTCACAAGTCAAATGGAAACCTTCCACTCAACTTCCTCCGAAGGCAATCCAAGCCCTTCTCAAGTCTCTACAACATCCG
AGTACCCATCACAACCTTCATCTCCACCCAACACACCACGCCAG**GGCGGCGGCGATTCTCTTGAATTTATTGCTAGTAAGCTTGCG**TAGTTACTTAAAAACATATTATCACA
AAAAGCCATGACCAACTTAAACAGAATCAAAATAAACTCTGGGGCAAATAACAATGGAGTTGCCAATCCTCAAAGCAAATGCAATTACCACAATCCTCGCTGCAGTCACATT
TTGCTTTGCTTCTGGT**GGCGGAGGAGGGGGC**CAAAACATCACTGAAGAATTTTATCAATCAACATGCAGTGCAGTTAGCAAAGGCTATCTTAGTGCTCTAAGAACTGGTTGG
TATACTAGTGTTATAACTATAGAATTAAGTAATATCAAGAAAAATAAGTGTAATGGAACAGATGCTAAGGTAAAATTGATGAAACAAGAATTAGATAAATATAAAAATGCTG
TAACAGAATTGCAGTTGCTCATGCAAAGCACACCAGCAGCAAACAATCGAGCCAGAAGAGAACTACCAAGGTTTATGAATTATACACTCAACAATACCAAAAAAACCAATGT
AACATTAAGCAAGAAAAGGAAAAGAAGATTTCTTGGTTTTTTGTTAGGTGTTGGATCTGCAATCGCCAGTGGCATTGCTGTATCTAAGGTCCTGCACTTAGAAGGAGAAGTG
AACAAGATCAAAAGTGCTCTACTATCCACAAACAAGGCCGTAGTCAGCTTATCAAATGGAGTTAGTGTCTTAACCAGCAGAGTGTTAGACCTCAAAAACTATATAGATAAAC
AATTGTTACCTATTGTGAATAAGCAAAGCTGCAGAATATCAAATATAGAAACTGTGATAGAGTTCCAACAAAAGAACAACAGACTACTAGAGATTACCAGGGAATTTAGTGT
TAATGCAGGTGTAACTACACCTGTAAGCACTTACATGTTAACTAATAGTGAATTATTGTCATTAATCAATGATATGCCTATAACAAATGATCAGAAAAAGTTAATGTCCAAC
AATGTTCAAATAGTTAGACAGCAAAGTTACTCTATCATGTCCATAATAAAAGAGGAAGTCTTAGCATATGTAGTACAATTACCACTATATGGTGTGATAGATACACCTTGTT
GGAAATTACACACATCCCCTCTATGTACAACCAACACAAAAGAAGGGTCAAACATCTGTTTAACAAGAACTGACAGAGGATGGTACTGTGACAATGCAGGATCAGTATCTTT
CTTCCCACAAGCTGAAAAATGTAAAGTTCAATCGAATCGAGTATTTTGTGACACAATGTACAGTTTAACATTACCAAGTGAAGTAAATCTCTGCAATGTTGACATATTCAAT

CCCAAATATGATTGTAAAATTATGACTTCAAAAACAGATGTAAGCAGCTCCGTTATCACATCTCTAGGAGCCATTGTGTCATGCTATGGCAAAACTAAATGTACAGCATCCA
ATAAAAATCGTGGAATCATAAAGACATTTTCTAACGGGTGTGATTATGTATCAAATAAAGGGGTGGACACTGTGTCTGTAGGTAACACATTATATTATGTAAATAAGCAAGA
AGGCAAAAGTCTCTATGTAAAAGGTGAACCAATAATAAATTTCTATGACCCATTAGTATTCCCCTCTGATGAATTTGATGCATCAATATCTCAAGTCAATGAGAAGATTAAC
CAGAGTTTAGCATTTATTCGTAAATCCGATGAATTATTACATAATGTAAATGCTGGTAAATCAACCACAAATATCATGATAACTACTATAATTATAGTGATTATAGTAATAT
TGTTATCATTAATTGCTGTTGGACTGCTCCTATACTGTAAGGCCAGAAGCACACCAATCACACTAAGCAAGGATCAACTGAGTGGTATAAATAATATTGCATTTAGTAACTG
AATAAAAATAGCACCTAATCATGTTCTTACAATGGTTTACTATCTGCTCATAGACAACCCATCTATCATTGGATTTCTTAAAATCTGAACTTCATCGAAACTCTTATCTAT
AAACCATCTCACTTACACTATTTAAGTAGATTCCTAGTTTATAGTTATATAAAACACAATTGAATGCCAGATTAACTTACCATCTGTAAAAATGAAAACTGGGGCAAATATG
TCACGAAGGAATCCTTGCAAATTTGAAATTCGAGGTCATTGCTTAAATGGTAAGAGGTGTCATTTTAGTCATAATTATTTTGAATGGCCACCCCATGCACTGCTTGTAAGAC
AAAACTTTATGTTAAACAGAATACTTAAGTCTATGGATAAAAGTATAGATACCTTATCAGAAATAAGTGGAGCTGCAGAGTTGGACAGAACAGAAGAGTATGCTCTTGGTGT
AGTTGGAGTGCTAGAGAGTTATATAGGATCAATAAACAATATAACTAAACAATCAGCATGTGTTGCCATGAGCAAACTCCTCACTGAACTCAATAGTGATGATATCAAAAAG
CTGAGGGACAATGAAGAGCTAAATTCACCCAAGATAAGAGTGTACAATACTGTCATATCATATATTGAAAGCAACAGGAAAAACAATAAACAAACTATCCATCTGTTAAAAA
GATTGCCAGCAGACGTATTGAAGAAAACCATCAAAAACACATTGGATATCCATAAGAGCATAACCATCAACAACCCAAAAGAATCAACTGTTAGTGATACAAATGACCATGC
CAAAAATAATGATACTACCTGACAAATATCCTTGTAGTATAACTTCCATACTAATAACAAGTAGATGTAGAGTTACTATGTATAATCAAAAGAACACACTATATTTCAATCA
AAACAACCCAAATAACCATATGTACTCACCGAATCAAACATTCAATGAAATCCATTGGACCTCTCAAGAATTGATTGACACAATTCAAATTTTTCTACAACATCTAGGTATT
ATTGAGGATATATATACAATATATATATTAGTGTCATAACACTCAATTCTAACACTCACCACATCGTTACATTATTAATTCAAACAATTCAAGTTGTGGGACAAAATGGATC
CCATTATTAATGGAAATTCTGCTAATGTTTATCTAACCGATAGTTATTTAAAAGGTGTTATCTCTTTCTCAGAGTGTAATGCTTTAGGAAGTTACATATTCAATGGTCCTTA
TCTCAAAAATGATTATACCAACTTAATTAGTAGACAAAATCCATTAATAGAACACATGAATCTAAAGAAACTAAATATAACACAGTCCTTAATATCTAAGTATCATAAAGGT
GAAATAAAATTAGAAGAACCTACTTATTTTCAGTCATTACTTATGACATACAAGAGTATGACCTCGTCAGAACAGATTGCTACCACTAATTTACTTAAAAAGATAATAAGAA
GAGCTATAGAAATAAGTGATGTCAAAGTCTATGCTATATTGAATAAACTAGGGCTTAAAGAAAAGGACAAGATTAAATCCAACAATGGACAAGATGAAGACAACTCAGTTAT
TACGACCATAATCAAAGATGATATACTTTCAGCTGTTAAAGATAATCAATCTCATCTTAAAGCAGACAAAAATCACTCTACAAAACAAAAAGACACAATCAAAACAACACTC
TTGAAGAAATTGATGTGTTCAATGCAACATCCTCCATCATGGTTAATACATTGGTTTAACTTATACACAAAATTAAACAACATATTAACACAGTATCGATCAAATGAGGTAA
AAAACCATGGGTTTACATTGATAGATAATCAAACTCTTAGTGGATTTCAATTTATTTTGAACCAATATGGTTGTATAGTTTATCATAAGGAACTCAAAAGAATTACTGTGAC
AACCTATAATCAATTCTTGACATGGAAAGATATTAGCCTTAGTAGATTAAATGTTTGTTTAATTACATGGATTAGTAACTGCTTGAACACATTAAATAAAAGCTTAGGCTTA
AGATGCGGATTCAATAATGTTATCTTGACACAACTATTCCTTTATGGAGATTGTATACTAAAGCTATTTCACAATGAGGGGTTCTACATAATAAAAGAGGTAGAGGGATTTA
TTATGTCTCTAATTTTAAATATAACAGAAGAAGATCAATTCAGAAAACGATTTTATAATAGTATGCTCAACAACATCACAGATGCTGCTAATAAAGCTCAGAAAAATCTGCT
ATCAAGAGTATGTCATACATTATTAGATAAGACAGTGTCCGATAATATAATAAATGGCAGATGGATAATTCTATTAAGTAAGTTCCTTAAATTAATTAAGCTTGCAGGTGAC
AATAACCTTAACAATCTGAGTGAACTATATTTTTTGTTCAGAATATTTGGACACCCAATGGTAGATGAAAGACAAGCCATGGATGCTGTTAAAATTAATTGCAATGAGACCA
AATTTTACTTGTTAAGCAGTCTGAGTATGTTAAGAGGTGCCTTTATATATAGAATTATAAAAGGGTTTGTAAATAATTACAACAGATGGCCTACTTTAAGAAATGCTATTGT
TTTACCCTTAAGATGGTTAACTTACTATAAACTAAACACTTATCCTTCTTTGTTGGAACTTACAGAAAGAGATTTGATTGTGTTATCAGGACTACGTTTCTATCGTGAGTTT
CGGTTGCCTAAAAAAGTGGATCTTGAAATGATTATAAATGATAAAGCTATATCACCTCCTAAAAATTTGATATGGACTAGTTTCCCTAGAAATTACATGCCATCACACATAC
AAAACTATATAGAACATGAAAAATTAAAATTTTCCGAGAGTGATAAATCAAGAAGAGTATTAGAGTATTATTTAAGAGATAACAAATTCAATGAATGTGATTTATACAACTG
TGTAGTTAATCAAAGTTATCTCAACAACCCTAATCATGTGGTATCATTGACAGGCAAAGAAAGAGAACTCAGTGTAGGTAGAATGTTTGCAATGCAACCGGGAATGTTCAGA
CAGGTTCAAATATTGGCAGAGAAAATGATAGCTGAAAACATTTTACAATTCTTTCCTGAAAGTCTTACAAGATATGGTGATCTAGAACTACAAAAAATATTAGAATTGAAAG
CAGGAATAAGTAACAAATCAAATCGCTACAATGATAATTACAACAATTACATTAGTAAGTGCTCTATCATCACAGATCTCAGCAAATTCAATCAAGCATTTCGATATGAAAC
GTCATGTATTTGTAGTGATGTGCTGGATGAACTGCATGGTGTACAATCTCTATTTTCCTGGTTACATTTAACTATTCCTCATGTCACAATAATATGCACATATAGGCATGCA
CCCCCCTATATAGGAGATCATATTGTAGATCTTAACAATGTAGATGAACAAAGTGGATTATATAGATATCACATGGGTGGCATCGAAGGGTGGTGTCAAAAACTGTGGACCA
TAGAAGCTATATCACTATTGGATCTAATATCTCTCAAAGGGAAATTCTCAATTACTGCTTTAATTAATGGTGACAATCAATCAATAGATATAAGCAAACCAATCAGACTCAT
GGAAGGTCAAACTCATGCTCAAGCAGATTATTTGCTAGCATTAAATAGCCTTAAATTACTGTATAAAGAGTATGCAGGCATAGGCCACAAATTAAAAGGAACTGAGACTTAT
ATATCACGAGATATGCAATTTATGAGTAAAACAATTCAACATAACGGTGTATATTACCCAGCTAGTATAAAGAAAGTCCTAAGAGTGGGACCGTGGATAAACACTATACTTG
ATGATTTCAAAGTGAGTCTAGAATCTATAGGTAGTTTGACACAAGAATTAGAATATAGAGGTGAAAGTCTATTATGCAGTTTAATATTTAGAAATGTATGGTTATATAATCA
GATTGCTCTACAATTAAAAAATCATGCATTATGTAACAATAAACTATATTTGGACATATTAAAGGTTCTGAAACACTTAAAAACCTTTTTTAATCTTGATAATATTGATACA
GCATTAACATTGTATATGAATTTACCCATGTTATTTGGTGGTGGTGATCCCAACTTGTTATATCGAAGTTTCTATAGAAGAACTCCTGACTTCCTCACAGAGGCTATAGTTC
ACTCTGTGTTCATACTTAGTTATTATACAAACCATGACTTAAAAGATAAACTTCAAGATCTGTCAGATGATAGATTGAATAAGTTCTTAACATGCATAATCACGTTTGACAA
AAACCCTAATGCTGAATTCGTAACATTGATGAGAGATCCTCAAGCTTTAGGGTCTGAGAGACAAGCTAAAATTACTAGCGAAATCAATAGACTGGCAGTTACAGAGGTTTTG
AGTACAGCTCCAAACAAAATATTCTCCAAAAGTGCACAACATTATACTACTACAGAGATAGATCTAAATGATATTATGCAAAATATAGAACCTACATATCCTCATGGGCTAA
GAGTTGTTTATGAAAGTTTACCCTTTTATAAAGCAGAGAAAATAGTAAATCTTATATCAGGTACAAAATCTATAACTAACATACTGGAAAAAACTTCTGCCATAGACTTAAC
AGATATTGATAGAGCCACTGAGATGATGAGGAAAAACATAACTTTGCTTATAAGGATACTTCCATTGGATTGTAACAGAGATAAAAGAGAGATATTGAGTATGGAAAACCTA
AGTATTACTGAATTAAGCAAATATGTTAGGGAAAGATCTTGGTCTTTATCCAATATAGTTGGTGTTACATCACCCAGTATCATGTATACAATGGACATCAAATATACTACAA
GCACTATATCTAGTGGCATAATTATAGAGAAATATAATGTTAACAGTTTAACACGTGGTGAGAGAGGACCCACTAAACCATGGGTTGGTTCATCTACACAAGAGAAAAAAAC
AATGCCAGTTTATAATAGACAAGTCTTAACCAAAAAACAGAGAGATCAAATAGATCTATTAGCAAAATTGGATTGGGTGTATGCATCTATAGATAACAAGGATGAATTCATG
GAAGAACTCAGCATAGGAACCCTTGGGTTAACATATGAAAAGGCCAAGAAATTATTTCCACAATATTTAAGTGTCAATTATTTGCATCGCCTTACAGTCAGTAGTAGACCAT
GTGAATTCCCTGCATCAATACCAGCTTATAGAACAACAAATTATCACTTTGACACTAGCCCTATTAATCGCATATTAACAGAAAAGTATGGTGATGAAGATATTGACATAGT
ATTCCAAAACTGTATAAGCTTTGGCCTTAGTTTAATGTCAGTAGTAGAACAATTTACTAATGTATGTCCTAACAGAATTATTCTCATACCTAAGCTTAATGAGATACATTTG
ATGAAACCTCCCATATTCACAGGTGATGTTGATATTCACAAGTTAAAACAAGTGATACAAAAACAGCATATGTTTTTACCAGACAAAATAAGTTTGACTCAATATGTGGAAT
TATTCTTAAGTAATAAAACACTCAAATCTGGATCTCATGTTAATTCTAATTTAATATTGGCACATAAAATATCTGACTATTTTCATAATACTTACATTTTAAGTACTAATTT
AGCTGGACATTGGATTCTGATTATACAACTTATGAAAGATTCTAAAGGTATTTTTGAAAAAGATTGGGGAGAGGGATATATAACTGATCATATGTTTATTAATTTGAAAGTT
TTCTTCAATGCTTATAAGACCTATCTCTTGTGTTTTCATAAAGGTTATGGCAAAGCAAAGCTGGAGTGTGATATGAACACTTCAGATCTTCTATGTGTATTGGAATTAATAG
ACAGTAGTTATTGGAAGTCTATGTCTAAGGTATTTTTAGAACAAAAAGTTATCAAATACATTCTTAGCCAAGATGCAAGTTTACATAGAGTAAAAGGATGTCATAGCTTCAA
ATTATGGTTTCTTAAACGTCTTAATGTAGCAGAATTCACAGTTTGCCCTTGGGTTGTTAACATAGATTATCATCCAACACATATGAAAGCAATATTAACTTATATAGATCTT
GTTAGAATGGGATTGATAAATATAGATAGAATACACATTAAAAATAAACACAAATTCAATGATGAATTTTATACTTCTAATCTCTTCTACATTAATTATAACTTCTCAGATA
ATACTCATCTATTAACTAAACATATAAGGATTGCTAATTCTGAATTAGAAAATAATTACAACAAATTATATCATCCTACACCAGAAACCCTAGAGAATATACTAGCCAATCC
GATTAAAAGTAATGACAAAAAGACACTGAATGACTATTGTATAGGTAAAAATGTTGACTCAATAATGTTACCATTGTTATCTAATAAGAAGCTTATTAAATCGTCTGCAATG
ATTAGAACCAATTACAGCAAACAAGATTTGTATAATTTATTCCCTATGGTTGTGATTGATAGAATTATAGATCATTCAGGCAATACAGCCAAATCCAACCAACTTTACACTA
CTACTTCCCACCAAATATCTTTAGTGCACAATAGCACATCACTTTACTGCATGCTTCCTTGGCATCATATTAATAGATTCAATTTTGTATTTAGTTCTACAGGTTGTAAAAT
TAGTATAGAGTATATTTTAAAAGATCTTAAAATTAAAGATCCCAATTGTATAGCATTCATAGGTGAAGGAGCAGGGAATTTATTATTGCGTACAGTAGTGGAACTTCATCCT
GACATAAGATATATTTACAGAAGTCTGAAAGATTGCAATGATCATAGTTTACCTATTGAGTTTTTAAGGCTGTACAATGGACATATCAACATTGATTATGGTGAAAATTTGA
CCATTCCTGCTACAGATGCAACCAACAACATTCATTGGTCTTATTTACATATAAAGTTTGCTGAACCTATCAGTCTTTTTGTCTGTGATGCCGAATTGTCTGTAACAGTCAA
CTGGAGTAAAATTATAATAGAATGGAGCAAGCATGTAAGAAAGTGCAAGTACTGTTCCTCAGTTAATAAATGTATGTTAATAGTAAAATATCATGCTCAAGATGATATTGAT
TTCAAATTAGACAATATAACTATATTAAAAACTTATGTATGCTTAGGCAGTAAGTTAAAGGGATCGGAGGTTTACTTAGTCCTTACAATAGGTCCTGCGAATATATTCCCAG
TATTTAATGTAGTACAAAATGCTAAATTGATACTATCAAGAACCAAAAATTTCATCATGCCTAAGAAAGCTGATAAAGAGTCTATTGATGCAAATATTAAAAGTTTGATACC
CTTTCTTTGTTACCCTATAACAAAAAAAGGAATTAATACTGCATTGTCAAAACTAAAGAGTGTTGTTAGTGGAGATATACTATCATATTCTATAGCTGGACGTAATGAAGTT
TTCAGCAATAAACTTATAAATCATAAGCATATGAACATCTTAAAATGGTTCAATCATGTTTTAAATTTCAGATCAACAGAACTAAACTATAACCATTTATATATGGTAGAAT
CTACATATCCTTACCTAAGTGAATTGTTAAACAGCTTGACAACCAATGAACTTAAAAAACTGATTAAAATCACAGGTAGTCTGTTATACAACTTTCATAATGAATAATGAAT
AAAGATCTTATAATAAAAATTCCCATAGCTATACACTAACACTGTATTCAATTATAGTTATTAAAAATTAAAAATCATATAATTTTTAAATAACTTTTAGTGAACTAATCC
TAAAGTTATCATTTTAATCTTGGAGGAATAAATTTAAACCCTAATCTAATTGGTTTATATGTGTATTAACTAAATTACGAGATATTAGTTTTGACACTTTTTTTCTCGT

Antibody sequences

Signal sequence / VH/VL / CH/CL / linker / ybbR-tag / affinity tag

CR9501 HC:
MGWSCIILFLVATATGVHSQVQLVQSGPGLVKPSQTLALTCTVSGASINSDNYYWTWIRQRPGGGLEWIGHISYTGNTYYTPSLKSRLSMSLETSQSQF
SLRLTSVTAADSAVYFCAACGAYVLISNCGWFDSWGQGTQVTVSSASTKGPSVFPLAPSSKSTSGGTAALGCLVKDYFPEPVTVSWNSGALTSGVHTFP
AVLQSSGLYSLSSVVTVPSSSLGTQTYICNVNHKPSNTKVDKRVEPKSCDKTHTCPPCPAPELLGGPSVFLFPPKPKDTLMISRTPEVTCVVVDVSHED
PEVKFNWYVDGVEVHNAKTKPREEQYNSTYRVVSVLTVLHQDWLNGKEYKCKVSNKALPAPIEKTISKAKGQPREPQVYTLPPSRDELTKNQVSLTCLV
KGFYPSDIAVEWESNGQPENNYKTTPPVLDSDGSFFLYSKLTVDKSRWQQGNVFSCSVMHEALHNHYTQKSLSLSPGKGGSGGSDSLEFIASKLA*

CR9501 LC:
MGWSCIILFLVATATGVHSEIVMTQSPSSLSASIGDRVTITCQASQDISTYLNWYQQKPGQAPRLLIYGASNLETGVPSRFTGSGYGTDFSVTISSLQP
EDIATYYCQQYQYLPYTFAPGTKVEIKRTVAAPSVFIFPPSDEQLKSGTASVVCLLNNFYPREAKVQWKVDNALQSGNSQESVTEQDSKDSTYSLSSTL
TLSKADYEKHKVYACEVTHQGLSSPVTKSFNRGEC

5C4 HC:
MGWSCIILFLVATATGVHSVQLQQSGAELVKPGASVKLSCTASGFNIKDTFFHWVKQRPEQGLEWIGRIDPADGHTKYDPKFQGKATITADTSSNTAFL
QLSSLTSVDTAVYYCATTITAVVPTPYNAMDYWGQGTTVTVSSASTKGPSVFPLAPSSKSTSGGTAALGCLVKDYFPEPVTVSWNSGALTSGVHTFPAV
LQSSGLYSLSSVVTVPSSSLGTQTYICNVNHKPSNTKVDKRVEPKSCDKTHTCPPCPAPELLGGPSVFLFPPKPKDTLMISRTPEVTCVVVDVSHEDPE
VKFNWYVDGVEVHNAKTKPREEQYNSTYRVVSVLTVLHQDWLNGKEYKCKVSNKALPAPIEKTISKAKGQPREPQVYTLPPSRDELTKNQVSLTCLVKG
FYPSDIAVEWESNGQPENNYKTTPPVLDSDGSFFLYSKLTVDKSRWQQGNVFSCSVMHEALHNHYTQKSLSLSPGKGGSGGSDSLEFIASKLA*

5C4 LC:
MGWSCIILFLVATATGVHSDIVLTQSPASLAVSLGQRTTISCRASESVDSFDNSFIHWYQQKPGQPPKLLIFLASSLESGVPARFSGSGSRTDFTLTID
PVEADDAATYYCQQSNEDPFTFGSGTKLEIKRADAAPSVFIFPPSDEQLKSGTASVVCLLNNFYPREAKVQWKVDNALQSGNSQESVTEQDSKDSTYSL
SSTLTLSKADYEKHKVYACEVTHQGLSSPVTKSFNRGEC*

Motavizumab HC:
MGWSCIILFLVATATGVHSQVTLRESGPALVKPTQTLTLTCTFSGFSLSTAGMSVGWIRQPPGKALEWLADIWWDDKKHYNPSLKDRLTISKDTSKNQV
VLKVTNMDPADTATYYCARDMIFNFYFDVWGQGTTVTVSSASTKGPSVFPLAPSSKSTSGGTAALGCLVKDYFPEPVTVSWNSGALTSGVHTFPAVLQS
SGLYSLSSVVTVPSSSLGTQTYICNVNHKPSNTKVDKRVEPKSCDKTHTCPPCPAPELLGGPSVFLFPPKPKDTLMISRTPEVTCVVVDVSHEDPEVKF
NWYVDGVEVHNAKTKPREEQYNSTYRVVSVLTVLHQDWLNGKEYKCKVSNKALPAPIEKTISKAKGQPREPQVYTLPPSRDELTKNQVSLTCLVKGFYP
SDIAVEWESNGQPENNYKTTPPVLDSDGSFFLYSKLTVDKSRWQQGNVFSCSVMHEALHNHYTQKSLSLSPGKGGSGGSDSLEFIASKLA*

Motavizumab LC:
MGWSCIILFLVATATGVHSDIQMTQSPSTLSASVGDRVTITCSASSRVGYMHWYQQKPGKAPKLLIYDTSKLASGVPSRFSGSGSGTEFTLTISSLQPD
DFATYYCFQGSGYPFTFGGGTKVEIKRTVAAPSVFIFPPSDEQLKSGTASVVCLLNNFYPREAKVQWKVDNALQSGNSQESVTEQDSKDSTYSLSSTLT
LSKADYEKHKVYACEVTHQGLSSPVTKSFNRGEC*

101F HC:
MGWSCIILFLVATATGVHSQVTLKESGPGILQPSQTLSLTCSFSGFSLSTSGMGVSWIRQPSGKGLEWLAHIYWDDDKRYNPSLKSRLTISKDTSRNQV
FLKITSVDTADTATYYCARLYGFTYGFAYWGQGTLVTVSSASTKGPSVFPLAPSSKSTSGGTAALGCLVKDYFPEPVTVSWNSGALTSGVHTFPAVLQS
SGLYSLSSVVTVPSSSLGTQTYICNVNHKPSNTKVDKRVEPKSCDKTHTCPPCPAPELLGGPSVFLFPPKPKDTLMISRTPEVTCVVVDVSHEDPEVKF
NWYVDGVEVHNAKTKPREEQYNSTYRVVSVLTVLHQDWLNGKEYKCKVSNKALPAPIEKTISKAKGQPREPQVYTLPPSRDELTKNQVSLTCLVKGFYP
SDIAVEWESNGQPENNYKTTPPVLDSDGSFFLYSKLTVDKSRWQQGNVFSCSVMHEALHNHYTQKSLSLSPGKGGSGGSDSLEFIASKLA*

101F LC:
MGWSCIILFLVATATGVHSDIVLTQSPASLAVSLGQRATIFCRASQSVDYNGISYMHWFQQKPGQPPKLLIYAASNPESGIPARFTGSGSGTDFTLNIH
PVEEEDAATYYCQQIIEDPWTFGGGTKLEIKRADAAPSVFIFPPSDEQLKSGTASVVCLLNNFYPREAKVQWKVDNALQSGNSQESVTEQDSKDSTYSL
SSTLTLSKADYEKHKVYACEVTHQGLSSPVTKSFNRGEC*

ADI-19425 HC:
MGWSCIILFLVATATGVHSEVQLVESGGGLVKPGGSLRLSCAASGFTFSSYSMNWVRQAPGKGLEWVSSISSSSSYIYYADSVKGRFTISRDNAKNSLY
LQMNSLRAEDTAVYYCARLGYCSGGSCHFDYWGQGTLVTVSSASTKGPSVFPLAPSSKSTSGGTAALGCLVKDYFPEPVTVSWNSGALTSGVHTFPAVL
QSSGLYSLSSVVTVPSSSLGTQTYICNVNHKPSNTKVDKRVEPKSCDKTHTCPPCPAPELLGGPSVFLFPPKPKDTLMISRTPEVTCVVVDVSHEDPEV
KFNWYVDGVEVHNAKTKPREEQYNSTYRVVSVLTVLHQDWLNGKEYKCKVSNKALPAPIEKTISKAKGQPREPQVYTLPPSRDELTKNQVSLTCLVKGF
YPSDIAVEWESNGQPENNYKTTPPVLDSDGSFFLYSKLTVDKSRWQQGNVFSCSVMHEALHNHYTQKSLSLSPGKGGSGGSDSLEFIASKLA*

ADI-19425 LC:
MGWSCIILFLVATATGVHSQPVLTQPPSVSGAPGQRVTISCTGSSSNIGAGYDVHWYQQLPGTAPKLLIYGNSNRPSGVPDRFSGSKSGTSASLAITGL
QAEDEADYYCQSYDSSLSGFYVFGTGTKLTVLGQPKAAPSVFIFPPSDEQLKSGTASVVCLLNNFYPREAKVQWKVDNALQSGNSQESVTEQDSKDSTY
SLSSTLTLSKADYEKHKVYACEVTHQGLSSPVTKSFNRGEC*

ADI-14353 HC:
MGWSCIILFLVATATGVHSQVQLVQSGAEVKKPGSSVKVSCKASGGTFSSYTISWVRQAPGQGLEWMGRIKPIIGIANNAQRFKGRVTITAEKSTGTAY
MELSSLTSEDTAVYYCARGGYDYYGMDVWGQGTTVTVSSASTKGPSVFPLAPSSKSTSGGTAALGCLVKDYFPEPVTVSWNSGALTSGVHTFPAVLQSS
GLYSLSSVVTVPSSSLGTQTYICNVNHKPSNTKVDKRVEPKSCDKTHTCPPCPAPELLGGPSVFLFPPKPKDTLMISRTPEVTCVVVDVSHEDPEVKFN

WYVDGVEVHNAKTKPREEQYNSTYRVVSVLTVLHQDWLNGKEYKCKVSNKALPAPIEKTISKAKGQPREPQVYTLPPSRDELTKNQVSLTCLVKGFYPS
DIAVEWESNGQPENNYKTTPPVLDSDGSFFLYSKLTVDKSRWQQGNVFSCSVMHEALHNHYTQKSLSLSPGKGGSGGSDSLEFIASKLA*

ADI-14353 LC:
MGWSCIILFLVATATGVHSQSALTQPASVSGSPGQSITISCTGTSSDVGGYNYVSWHQQHPGKAPKLLIYDVSNRPSGVSNRFSGSKSGNTASLSISGL
QAEDEADYYCSSFTSTSTPYVFGTGTQLTVLGQPKAAPSVFIFPPSDEQLKSGTASVVCLLNNFYPREAKVQWKVDNALQSGNSQESVTEQDSKDSTYS
LSSTLTLSKADYEKHKVYACEVTHQGLSSPVTKSFNRGEC*

ADI-14359 HC:
MGWSCIILFLVATATGVHSQVTLRESGPALVKPTQTLTLTCTFSGFSLSTSGMCVSWIRQPPGKALEWLARIDWDDDKYYSTSLKTRLTISKDTSKNQV
VLTMTNMDPVDTATYYCARATNYDSSGYYSLYFDYWGQGTLVTVSSASTKGPSVFPLAPSSKSTSGGTAALGCLVKDYFPEPVTVSWNSGALTSGVHTF
PAVLQSSGLYSLSSVVTVPSSSLGTQTYICNVNHKPSNTKVDKRVEPKSCDKTHTCPPCPAPELLGGPSVFLFPPKPKDTLMISRTPEVTCVVVDVSHE
DPEVKFNWYVDGVEVHNAKTKPREEQYNSTYRVVSVLTVLHQDWLNGKEYKCKVSNKALPAPIEKTISKAKGQPREPQVYTLPPSRDELTKNQVSLTCL
VKGFYPSDIAVEWESNGQPENNYKTTPPVLDSDGSFFLYSKLTVDKSRWQQGNVFSCSVMHEALHNHYTQKSLSLSPGKGGSGGSDSLEFIASKLA*

ADI-14359 LC:
MGWSCIILFLVATATGVHSDIQMTQSPSSLSASVGDRVTITCRASQSISSYLNWYQQKPGKAPKLLIYAASSLQSGVPSRFSGSGSGTDFTLTISSLQP
EDFATYYCQQSYSTPYTFGGGTKVEIKRTVAAPSVFIFPPSDEQLKSGTASVVCLLNNFYPREAKVQWKVDNALQSGNSQESVTEQDSKDSTYSLSSTL
TLSKADYEKHKVYACEVTHQGLSSPVTKSFNRGEC*

3D3 HC:
MGWSCIILFLVATATGVHSEEQLVESGGGLVQPGRSLRLSCVGSGLRFEEHAMHWVRQAPGRGLEWVSGISWNSGSVGYADSVKGRFTTSRDNAKDILF
LEMNTLRSEDTALYFCAIMVATTKNDFHYYKDVWGKGTTVTVSSASTKGPSVFPLAPSSKSTSGGTAALGCLVKDYFPEPVTVSWNSGALTSGVHTFPA
VLQSSGLYSLSSVVTVPSSSLGTQTYICNVNHKPSNTKVDKRVEPKSCDKTHTCPPCPAPELLGGPSVFLFPPKPKDTLMISRTPEVTCVVVDVSHEDP
EVKFNWYVDGVEVHNAKTKPREEQYNSTYRVVSVLTVLHQDWLNGKEYKCKVSNKALPAPIEKTISKAKGQPREPQVYTLPPSRDELTKNQVSLTCLVK
GFYPSDIAVEWESNGQPENNYKTTPPVLDSDGSFFLYSKLTVDKSRWQQGNVFSCSVMHEALHNHYTQKSLSLSPGKGGSGGSDSLEFIASKLA*

3D3 LC:
MGWSCIILFLVATATGVHSQIVLTQSPATLSLSPGERATLSCRASQSVSNHLAWYQQKPGQAPRLLIYETSNRATGIPPRFSGSGSGTDFTLTISSLEP
EDFAVYYCQQRNNWYTFGQGTKLEIKRTVAAPSVFIFPPSDEQLKSGTASVVCLLNNFYPREAKVQWKVDNALQSGNSQESVTEQDSKDSTYSLSSTLT
LSKADYEKHKVYACEVTHQGLSSPVTKSFNRGEC*

5C4 IgM HC:
MGWSCIILFLVATATGVHSVQLQQSGAELVKPGASVKLSCTASGFNIKDTFFHWVKQRPEQGLEWIGRIDPADGHTKYDPKFQGKATITADTSSNTAFL
QLSSLTSVDTAVYYCATTITAVVPTPYNAMDYWGQGTTVTVSSASASAPTLFPLVSCENSPSDTSSVAVGCLAQDFLPDSITFSWKYKNNSDISSTRGF
PSVLRGGKYAATSQVLLPSKDVMQGTDEHVVCKVQHPNGNKEKNVPLPVIAELPPKVSVFVPPRDGFFGNPRKSKLICQATGFSPRQIQVSWLREGKQV
GSGVTTDQVQAEAKESGPTTYKVTSTLTIKESDWLGQSMFTCRVDHRGLTFQQNASSMCVPDQDTAIRVFAIPPSFASIFLTKSTKLTCLVTDLTTYDS
VTISWTRQNGEAVKTHTNISESHPNATFSAVGEASICEDDWNSGERFTCTVTHTDLPSPLKQTISRPKGVALHRPDVYLLPPAREQLNLRESATITCLV
TGFSPADVFVQWMQRGQPLSPEKYVTSAPMPEPQAPGRYFAHSILTVSEEEWNTGETYTCVVAHEALPNRVTERTVDKSTGKPTLYNVSLVMSDTAGTC
YGGSHHHHHHGGSDSLEFIASKLA*

J Chain:
MGWSCIILFLVATATGVHSQEDERIVLVDNKCKCARITSRIIRSSEDPNEDIVERNIRIIVPLNNRENISDPTSPLRTRFVYHLSDLCKKCDPTEVELD
NQIVTATQSNICDEDSATETCYTYDRNKCYTAVVPLVYGGETKMVETALTPDACYPDGGSSGSNWSHPQFEKGGGGSNWSHPQFEK*

ADI-14359 Fab HC:
MGWSCIILFLVATATGVHSQVTLRESGPALVKPTQTLTLTCTFSGFSLSTSGMCVSWIRQPPGKALEWLARIDWDDDKYYSTSLKTRLTISKDTSKNQV
VLTMTNMDPVDTATYYCARATNYDSSGYYSLYFDYWGQGTLVTVSSASTKGPSVFPLAPSSKSTSGGTAALGCLVKDYFPEPVTVSWNSGALTSGVHTF
PAVLQSSGLYSLSSVVTVPSSSLGTQTYICNVNHKPSNTKVDKRVEPKSCDKGGSHHHHHHGGSDSLEFIASKLA*

D25 Fab HC:
MGWSCIILFLVATATGVHSQVQLVQSGAEVKKPGSSVMVSCQASGGPLRNYIINWLRQAPGQGPEWMGGIIPVLGTVHYAPKFQGRVTITADESTDTAY
IHLISLRSEDTAMYYCATETALVVSTTYLPHYFDNWGQGTLVTVSSASTKGPSVFPLAPSSKSTSGGTAALGCLVKDYFPEPVTVSWNSGALTSGVHTF
PAVLQSSGLYSLSSVVTVPSSSLGTQTYICNVNHKPSNTKVDKKVEPKSCDKGGSHHHHHHGGSDSLEFIASKLA*

D25 LC:
MGWSCIILFLVATATGVHSDIQMTQSPSSLSAAVGDRVTITCQASQDIVNYLNWYQQKPGKAPKLLIYVASNLETGVPSRFSGSGSGTDFSLTISSLQP
EDVATYYCQQYDNLPLTFGGGTKVEIKRTVAAPSVFIFPPSDEQLKSGTASVVCLLNNFYPREAKVQWKVDNALQSGNSQESVTEQDSKDSTYSLSSTL
TLSKADYEKHKVYACEVTHQGLSSPVTKSFNRGEC*
